# The Phosphates in Dental Hygiene

**Published:** 1863-09

**Authors:** Edward Parrish


### Selections.

THE PHOSPHATES IN DENTAL HYGIENE.—*By Edward Parrish.*—The great natural reservoir of the phosphates is in the igneous rocks, which contain phosphoric acid, combined with lime, magnesia, alumina and the oxides of several of the heavy metals, in minerals which are both numerous and widely diffused. Through the slow process of solution in the waters which permeate the crust of the earth, minute quantities of these generally insoluble salts are carried to the roots of plants, the delicate spongioles of which are continually drawing the fluid from the loosened soil to traverse the plant and be eliminated from its porous leaf surfaces. The mineral constituents being retained in the juices of the plant, are assimilated into its structure, and thus, through the vegetable food consumed by animals, are assimilated into its structure, and thus, through the vegetable food consumed by animals, are conveyed to the higher order of living beings, to the growth of which they are so essential. The importance of the supply of the mineral phosphates to the human system, is apparent from their presence as normal constituents of the blood, of the gastric juice, of the bones, and throughout the tissues generally, and their elimination in the urine and feces, so that any deficiency in the supply of these constituents must of consequence impair the health and vigor of the individual, and in early life especially, must interfere with growth and development.

This deficiency may occur from want of food containing a proper proportion of phosphatic salts, or from defective nutrition, both causes of common occurrence in a condition of society in which are found the half-fed poor, the pampered rich, the over-worked student and the dissipated idler, and in all conditions of life, the ignorant, the vicious and the negligent. Suitable and well-prepared food for all, though it would be a great desideratum, would not secure a normal condition without ample and well adjusted clothing, the avoidance of excesses, sufficient mastication of food, attention to personal cleanliness, free exercise in the open air and sunshine, and an observance of all the laws of health.

It is, in a great measure, by the neglect of these laws that the necessity is entailed upon the community, of doctors, and dentists and pharmaceutists; and it is to supply, by appropriate artificial means, those necessities of the system which grow out of defective hygiene, that the three kingdoms of nature are ransacked in the interest of materia medica. How, then, can the chemist supply the much needed phosphates to the weak and imperfectly developed? How can the dentist promote the growth of healthy teeth by supplying to the child at the period of dentition, the chief constituents of these important organs? This is the inquiry that it is the object of the present paper to answer.

An obstacle to the successful introduction of the artificially prepared mineral phosphates into the system, is found in their great insolubility, not only in water but in the digestive fluids—unless these are, at least, normally active. So that it is believed that precipitated phosphate of lime, one of the most common of these, when administered in powder, is liable to pass through the alimentary canal almost without diminution in quantity.

Prof. Jackson, of the University of Pennsylvania, was one of the first physicians to appreciate the advantage of eligible combinations of the phosphates in the proportions theoretically applicable to supply the waste in the system, and it was at his instance that some of the pharmaceutists of Philadelphia first attempted the application of chemical principles to this end. The first published recipe of the series which culminated in the so-called *chemical food*, was for a syrup of phosphate of lime; this was published in 1853, by Durand, Jr.; and was followed in the following year by the formula of T. S. Weigand, for a syrup of undissolved phosphate of lime, in which that salt, without excess of acid, was contained in a pulpy magma favorable to its absorption and assimilation, and an essay by Prof. Procter, indicating the methods of extemporaneous prescription best adapted to administer the several phosphates. As the demand for these combinations extended, other pharmaceutists, improving upon these formulæ, succeeded in combining in a liquid form the mixed phosphates, in the proportion indicated by Prof. Jackson; and in 1857 I furnished to the editor of the *American Journal of Pharmacy* a formula, which has since been pretty generally adopted, and has been found to give entire satisfaction when skillfully executed, not only as embodying the requisite materials, but also as constituting an elegant and agreeable pharmaceutical preparation.

It has long been a rule with some of the leading pharmaceutists, to introduce no new remedy which shall be unpleasant to the taste, and acting on this idea, the solution of phosphates is made into a syrup and colored a rich strawberry color by the aid of cochineal; the flavor adopted is that of orange-flower, and the acidity caused by the excess of phosphoric acid necessary to make a perfect solution, takes from the syrup the clawing sweetness, which is apt to become unpleasant to adults by long-continued use. The name adopted for this remedy has added greatly to its popularity; many persons who object to taking medicine, are attracted by the title *chemical food*, and finding benefit from its use, this prejudice is overcome. Each dose contains two and a half grains of phosphate of lime, which, according to recent investigations, is essential to the formation of cells, and which is an element of prime importance in dental hygiene, as a leading constituent of the teeth; one grain of iron, a very popular "hæmatogen," fractions of a grain of phosphate of soda and potassa, adapted to promote digestion, and to supply deficiencies in the secretions, and an excess of phosphoric acid, an admirable acid tonic.

In those cases where the indication is simply to supply phosphates of lime and iron in an assimilable form, free from an excess of acid, as in young children, whose tendencies are to deficient development of the osseous structures, the following extemporaneous preparation may be prescribed:

Chloride of Calcium, ℥iss.,

Phosphate of Soda, ℥vii,

Sulphate of Iron, ℨij,

Syrup of Ginger,

Water, of each, *f* ℥iv.

Triturate the chloride of calcium with the phosphate of soda, and three fluid ounces of the water, till the decomposition is complete, and a smooth mixture is obtained, then add the syrup, and finally the sulphate of iron, previously dissolved in a fluid ounce of the water. The resulting mixture consists of the hydrated phosphates of lime and iron, with inconsiderable amounts of sulphate of soda and common salt, rendered palatable by sugar, which always commends a medicine to the favor of children.

In my work on Pharmacy, now under revision for the third edition, I have given other forms of preparation of this series of salts; they have impressed me as worthy the attention of dentists, who meet with so many evidences of imperfect nutrition of the osseous system.

At the risk of being charged with exaggerating the merits of this class of nutritive tonics, I am induced to relate, in conclusion, a case mentioned to me some years ago, by Dr John H. Parrish, of Greensboro, Alabama, a physician of large experience and undoubted veracity. A colored slave on a plantation within his circuit of practice, had been long afflicted with a diseased bone of the leg, which having been under treatment, had grown so much worse of late, that he had determined to amputate it. The time was fixed, and the patient put under the use of *chemical food* as a preparatory tonic; meanwhile the doctor was taken ill with a hemorrhage from his lungs, followed by great prostration, which prevented his attention to practice for months. When he returned to his patient, he found him so far recovered as to be able to walk with facility, the discharges had nearly or quite ceased, his condemned limb was restored, and for aught we know, he may now come to the ownership of himself—*a whole man.*

